# Getah Virus (Alphavirus): An Emerging, Spreading Zoonotic Virus

**DOI:** 10.3390/pathogens11080945

**Published:** 2022-08-20

**Authors:** Bin Li, Huanyu Wang, Guodong Liang

**Affiliations:** 1 School of Public Health, Jiamusi University, Jiamusi 154007, China; 2 State Key Laboratory of Infectious Disease Prevention and Control, National Institute for Viral Disease Control and Prevention, Chinese Center for Disease Control and Prevention, Beijing 102206, China

**Keywords:** Getah virus, GETV, zoonotic disease, emerging infection, virus spreading, virus transmission, molecular genetic evolution, *Alphavirus*, *Togaviridae*

## Abstract

Getah virus (GETV) is a zoonotic virus transmitted by mosquitoes, belonging to the *Togaviridae* family, *Alphavirus* genus. It was first isolated from mosquitoes in Malaysia in 1955, being widespread in island countries in the South Pacific region. Since the beginning of the 21st century, GETV expanded its range and geographical distribution from low-latitude tropical regions to 60° north latitude, being isolated from 17 different species of mosquitoes belonging to five genera of Culicidae (*Culex*, *Anopheles*, *Armigeres*, *Aedes* and *Mansonia*), as well as from midges in Eurasia. Molecular genetic evolution analysis revealed large molecular differences between the mosquitoes currently circulating Eurasia and those in the South Pacific in 1950s. The number of disease outbreaks caused by GETV in animals is increasing alongside the types of animals infected, from horses and pigs to cattle, blue foxes and red pandas. The disease burden is severely underestimated, and the economic cost to livestock production remains unknown. Herein, we review GETV temporal and spatial distribution, molecular genetic evolution, transmission and data on disease outbreaks. This work provides a reference for public health workers engaged in GETV research and zoonotic disease prevention and control.

## 1. Introduction

Getah virus (GETV), a member of the *Alphavirus* genus of the *Togaviridae* family [1,2], was first isolated in 1955 from *Culex gelidus* mosquitoes collected from a rubber plantation in Malaysia [1]. Since then, different studies have found that GETV can be amplified in mosquitoes, transmitted to animals through bites, and virus-infected animals serve as a reservoir from which uninfected mosquitoes can acquire the virus. Thus, GETV undergoes a mosquito–vertebrate host–mosquito cycle in nature [1,2,3]. GETV infection causes disease in livestock with symptoms including fever, rash, oedema of the limbs and lymphadenopathy in horses and abortion in pigs [4,5]. Disease outbreaks in horses and pigs caused by GETV infection first occurred in Japan in the 1970s. Since then, several outbreaks have been reported in livestock and other animals in India [6], mainland China and so on [7].

GETV is an enveloped, single-stranded positive-strand RNA (ssRNA) virus [8]. The full-length viral genome is 11,000 to 12,000 nucleotides. The 5’ end has a methylated (7-methylguanosine) cap structure and the 3’ end has a variable number of poly (A) tails. The viral genome includes two open reading frames (ORFs). The first ORF is located in the first 2/3 of the 5’ end of the viral genome and encodes four viral non-structural proteins (nsP1–4), which are responsible for viral RNA transcription and replication. This region is followed by the 26 subgenomic promoter, which promotes the transcription of the intracellular subgenomic 26S RNA (subgenomic RNA) containing a second ORF. The second ORF, which is in frame in the final 1/3 at the 3’ end of the genome, encodes multiple viral structural proteins (C, E3, E2, 6K and E1) [9,10]. E2 is the main functional protein involved in infecting host cells, causing disease and triggering host immune responses [2,3].

Since the beginning of the 21st century, the geographical distribution of GETV has continually expanded, as has the frequency and area of animal disease outbreaks caused by GETV infection and the types of virus vectors and animals infected. GETV infection in animals is attracting the attention of virologists and animal public health workers. Herein, we review GETV temporal and spatial distribution, transmission vectors, molecular genetic evolution and infectious diseases in animals. This work provides a reference for public health workers engaged in the prevention and control of GETV and related infections.

## 2. Expansion of GETV in Geographical Region, Vectors and Host Animals

### 2.1. Literature Collection Method

The literature data of this study was derived from PubMed (https://pubmed.ncbi.nlm.nih.gov/ (accessed on 30 April 2022)) and CNKI (https://www.cnki.net (accessed on 30 April 2022)). According to the search rules of PubMed and CNKI, the search condition was set to (“Getah virus”) (Title/Abstract) in PubMed or (“Getah virus”) (Title) and (“Getah virus”) (Abstract) in CNKI. The retrieval time span was April 1969 to April 2022. After the retrieval, some studies with poor relevance to the research topic were removed through manual screening.

### 2.2. GETV Isolated in the 1950s and 1960s

**Figure 1:**
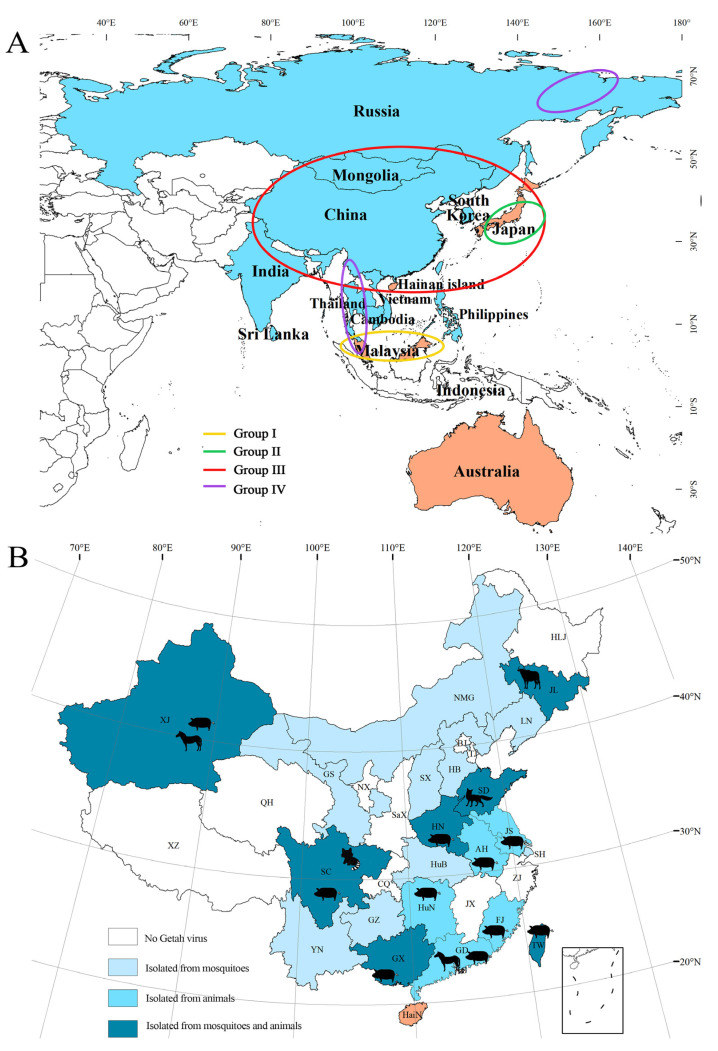
Geographical distribution of GETV: (**A**) Geographical distribution of GETV worldwide. (**B**) Geographical distribution of GETV in China. The orange area of (**A**) and (**B**) represents regions where GETV was present from 1955 to 1965. The light blue area of (**A**) is the current geographic distribution of GETV in Eurasia. The four colored circles (yellow, green, red and purple) represent the geographical distribution of GETV groups I, II, III and IV, respectively. Chinese provinces with animal host isolates are marked with different animal patterns on the figure (**B**). Chinese provinces: HLJ, Heilongjiang Province; JL, Jilin Province; LN, Liaoning Province; NMG, Neimenggu; XJ, Xinjiang; BJ, Beijing; TJ, Tianjin; HB, Hebei Province; SX, Shanxi Province; SaX, Shaanxi Province; GS, Gansu Province; QH, Qinghai Province; NX, Ningxia; SD, Shandong Province; SH, Shanghai; JS, Jiangsu Province; AH, Anhui Province; HeN, Henan Province; XZ, Xizang; ZJ, Zhejiang Province; JX, Jiangxi Province; HuB, Hubei Province; CQ, Chongqing; SC, Sichuan Province; HuN, Hunan Province; GZ, Guizhou Province; YN, Yunnan Province; FJ, Fujian Province; GD, Guangdong Province; GX, Guangxi; HaiN, Hainan; TW, Taiwan.

The first GETV (MM2021) was isolated from *Cx. gelidus* in Malaysia in October 1955 [1]. Subsequently, on 23 July 1956, a virus (M6/Mag-132) was isolated from *Cx. tritaeniorhynchus* collected from a pigpen 20 km north of Tokyo, Japan, and named Sagiyama virus (SAGV) [1]. The results of virus cross-neutralizing antibody titer detection and viral gene phylogenetic analysis showed that the M6/Mag-132 virus was an isolate of GETV in different regions [11,12]. In 1961, the GETV (N544) was isolated from Australia in the South Pacific [1], and GETV (M1) was isolated from a *Culex* species collected in Hainan Island, China, in 1964 [13]. In 1966, GETV was isolated from *Cx. tritaeniorhynchus* in Cambodia [1], which was the first time this virus was isolated from a mosquito vector in the southernmost region of Eurasia. Before the 1960s, GETV was mainly prevalent in different islands in the Pacific, then it began to spread to the southernmost parts of Eurasia (Figure 1).

### 2.3. Geographical Distribution of GETV

As of March 2022, there were 129 GETV strains listed in GenBank, and another 41 strains were found by searching Chinese and English references (these strains do not overlap with GenBank). Only 24 of these 169 GETV strains were isolated before 2000 (probably due to the limitations of sequencing technology); thus, the number of GETV isolates has increased rapidly since the beginning of the 21st century (Table S1).

The geographic distribution of GETV since 1955 spans from 1° north (Malaysia) to 60° north (Russia), and from 38° east to 140° east. As of March 2022, GETV had been isolated in 13 countries, including Indochina Peninsula countries in Asia (Vietnam, Thailand, Cambodia), Southeast Asian countries (Malaysia, the Philippines), the South Asian subcontinent (India, Sri Lanka), Australia, Japan, South Korea, Mongolia, China and Russia [14,15,16,17,18] (Table S1). Over almost 70 years (1955–2022), GETV has gradually spread from tropical to temperate regions, and it has even been found in the cold Arctic tundra [19]. GETV was first isolated in China in 1964, and by March 2022 it was found in 65% (22/34) of China’s provinces (Figure 1 and Table S1).

### 2.4. GETV Isolated from Bloodsucking Insects

Before 2000, GETV was only isolated from four species of *Culex* (Diptera: Culicidae) mosquitoes (*C. gelidus*, *Cx. tritaeniorhynchus*, *Cx. vishnui*, *Cx.* *fuscocephala*), including the first GETV isolated in Malaysia in 1955. By 2022, 113 of the 169 strains of GETV were isolated from bloodsucking insects, including five mosquito genera (17 species), 59 from *Culex* mosquito (36 from *Cx. tritaeniorhynchus*), 13 from *Armigeres*, 10 from *Anopheles*, 5 from *Aedes* and 2 from *Mansonia*, while the remaining 22 viruses were isolated from unclassified mosquitoes. In addition, there were two virus isolates from midges (*Culicoides* species). The number of species of bloodsucking insects known to carry and spread GETV has increased significantly. GETV strains isolated from bloodsucking insects are listed in Table S1.

### 2.5. GETV Isolated from Host Animals

As of March 2022, 56 strains of GETV had been isolated from various animal specimens, including pigs (37 strains), horses (12 strains), wild boars (2 strains), red pandas (2 strains), cattle (1 strain), blue fox (1 strain) and fox (1 strain). Host animal isolates were obtained from five countries (China, Japan, Korea, India and Thailand). In the past 10 years, 47 isolates have been acquired from animal specimens in 12 provinces in China, representing 84% (47/56) of the total number of animal isolates worldwide. GETV isolates of animal origin are listed in Table S1.

## 3. Animal Infection and Disease Outbreaks

### 3.1. Disease Outbreaks Caused by GETV in Animals

Although GETV was isolated from mosquitoes collected in places such as Malaysia and Japan in the 1950s, it was not until 1978 that the first outbreak of GETV-infected horses was identified in Japan [20]. Since then, GETV has reportedly caused many disease outbreaks in domesticated mammals including horses [6,21], pigs [5,7] and cattle [22] in Japan [4,5,20,21], India [6] and mainland China [7,22,23]. GETV can also cause disease outbreaks in domesticated blue foxes [23]. The prevalence of animal diseases caused by GETV infection is shown in Table 1.

#### 3.1.1. Horses

In 1978, 70 of the 455 bred racehorses at an equestrian training center in the Kanto region of Japan became ill. The clinical sign of illness detected in the infected horses included fever for one to four days, rash and oedema of the limbs for up to a week. The disease spread slowly throughout stables, gradually disappearing within 2 months [20]. An additional outbreak occurred in Ibaraki Prefecture the same year, with outbreaks of GETV infection in 722 of the 1903 horses at an equestrian training center [4]. Since then, in Japan [21,24,25], India [6] and China [26], disease outbreaks have occurred in GETV-infected horses many times (Table 1).

#### 3.1.2. Pigs

In 1985, outbreaks caused by GETV infection in pigs was first reported in Japan. Among the 12 piglets produced by one sow during the outbreak, 8 piglets developed symptoms including depression, tremors and yellowish-brown diarrhea 2 days after birth, and subsequently died within 3–5 days after birth. The remaining four piglets displayed hypoplasia [5]. In 2017, 1333 piglets from 2915 pigs (including 1000 sows) raised on a pig farm in Hunan Province, China, developed fever, anorexia, ataxia and tremors, and 200 piglets subsequently died. The outbreak also resulted in stillbirths or fetal mummies for 150 pregnant sows [7]. Pigs infected with GETV also suffered dysgenesis, severe diarrhea and death [10,27].

In 2018, 801 samples of pig lung, spleen, lymph node, intestinal segment, sow milk, boar semen and other samples were collected from 231 affected pig farms in four provinces of China (Henan, Hebei, Shanxi and Anhui) for GETV gene detection (Reverse Transcription-Polymerase Chain Reaction, RT-PCR) [28]. The results showed that the positive rate of pig farms was 13.9% (32/231), and the positive rate of specimens was 4.62% (37/801), indicating a relatively widespread GETV infection in pig herds in North China.

#### 3.1.3. Other Animals

In 2018, 10 out of 48 cattle raised on a cattle farm in Jilin Province in northeastern China developed fever, loss of appetite and depression, and test results revealed GETV infection [22]. In 2017, 25 (5-month-old) blue foxes raised in an animal farm in Shandong Province in eastern China developed fever, anorexia and depression, and 6 developed neurological symptoms then died on the third day of onset [23]. In 2020, GETV infection was found in dead red pandas *(Ailurus fulgens)* in Sichuan Province, China [29].

### 3.2. Seroepidemiological Analysis of GETV Infection in Animals 

Given the outbreaks of GETV in animals such as horses and pigs, seroepidemiological studies on animals infected with GETV have attracted attention. Japan, South Korea, India, China, Thailand, Indonesia and Malaysia have carried out a seroepidemiological investigation on GETV infection, among which Japan has carried out the most detailed surveys on horses, pigs and wild boars. Serum neutralization tests showed that the positive rate of GETV antibody in local horses in Japan before and after disease outbreaks was 6% (14/232) and 61.2% (172/282), respectively [30]. The positive rate for antibody in domestic pigs in many places in Japan ranged from 2.7% (1/37) to 19.1% (40/209) before and after the outbreak [31]. Results of multiple tests showed that the positive rate of antibody in local wild boars in Japan ranged from 3% (3/301) to 54.3% (39/70), significantly higher than in domestic pigs. Thus, wild boars may be a reservoir for amplification of GETV in nature [32].

GETV neutralizing antibodies (positive at a serum dilution of 1:10) were tested in five species of animals collected from Yunnan Province, southwestern China, and the results showed that the positive rates of neutralizing antibodies in serum samples from chicken, duck and dairy cattle were 2.2% (1/46), 5.6% (1/18) and 13.3% (2/15), respectively. The positive rates of neutralizing antibody in pigs and beef cattle serum samples were 45.9% (39/85) and 71.9% (23/32), respectively. Further test results showed that only low titers (1:10 to 1:20) of neutralizing antibodies against GETV could be detected in chickens, ducks and dairy cattle serum samples; by contrast, most pigs and beef cattle serum samples contained neutralizing antibodies at serum dilutions of 1:640 to 1280, accounting for 20.5% (8/39) and 60.9% (14/23) of the total number of positive specimens in these animals, respectively, and the neutralizing antibody titers of some specimens were even higher than 1:2560 [33]. The positive rates of antibody in enzyme-linked immunosorbent assays (ELISA) on sheep and goat specimens from Xinjiang in northwestern China were 11.7% (55/471) and 10.0% (47/308), respectively [34]. The positive rate for goats (detected by complement fixation test, CFT) in Hainan Province in 1982 was 37.5 (6/16) [13]. The above results suggest that GETV can infect a variety of animals, and horses, pigs (including wild boar) and beef cattle may be the sensitive hosts of GETV. The results of seroepidemiological investigation of animals infected with GETV are summarized in Table 2.

### 3.3. GETV Infections in Human

Serum samples from unknown fever patients and healthy individuals collected from 1979–1982 in Hainan Island, China, were subjected to seroepidemiological analysis of GETV. The results showed that 26.4% (24/91) of serum samples collected from local unknown fever patients in Hainan Island in June 1980 and May 1982 were positive for GETV antibodies (detected by CFT). Using the same detection method, 3.4% (2/58) of healthy human serum samples in 1979 and 1982 were found to be positive for GETV antibodies [13]. Detection of arbovirus antibodies in serum samples from healthy populations in New South Wales, Australia, was carried out in 1979. The results revealed positive GETV hemagglutination-inhibiting (HI) antibodies in the serum samples of local healthy people [45]. Among 737 healthy human serum samples collected in parts of Asia in the former Soviet Union between 1985 and 1988, 7% were positive for GETV HI antibodies [46]. Among 449 sera collected from residents of various age ranges in Sarawak, Malaysia, from 1961 to 1966, 135 samples were positive for neutralizing antibodies [47].

In countries of different latitudes, such as Australia, Malaysia, Hainan Island in China and the former Soviet Union, some healthy people and patients were found to be positive for GETV antibodies. However, as of 2022, there have been no reports of GETV infection causing human disease. There has also been no report of a positive viral gene test (RT-PCR) for specimens from fever patients. Since there have been many outbreaks of infectious diseases caused by the GETV in horses, pigs and cattle, special attention should be paid to the health monitoring of the personnel involved in animal care and treatment, humans being incidental hosts that become infected when encountering the enzootic cycle. GETV infection among these workers should be carefully examined, especially those presenting illnesses caused by viral infections, even mild illnesses from which people appear to full recover without treatment.

### 3.4. Methods for the Etiological Detection of GETV

As mentioned earlier, seroepidemiological detection methods for animals or humans infected with GETV can be detected by serological tests, such as a neutralization test (NT) [35], complement fixation test (CFT) [13], hemagglutination inhibition test (HIT) [31], enzyme-linked immunosorbent assay (ELISA) [32] and so on. ELISA is currently the most commonly used. There is no commercial antigen test method, so the etiological detection of GETV mainly includes virus gene amplification and virus isolation. Virus gene detection methods mainly include Reverse Transcription-Polymerase Chain Reaction (RT-PCR), listed as: (1) extracting the total RNA of the specimen and synthesizing the cDNA of the viral genome through reverse transcription (RT); (2) designing a pair of primers based on a certain region of the GETV gene, such as C and nsP3 regions; and (3) gene amplification is carried out using cDNA as a template. The nucleotide length of the amplified product of C gene fragment is 316 bp, and that of nsP3 is 810 bp [28]. Regarding virus isolation, bloodsucking insect specimens or animal specimens that can be collected are ground and centrifuged, and the supernatant is inoculated into tissue culture cells (BHK [47], VERO [5], ESK [5], SK-L [5], and HMLU-1 [5], RK-13 [17]).

## 4. Molecular Evolution of GETV

### 4.1. Group III GETV Is an Emerging, Dominant Virus Group

**Figure 2:**
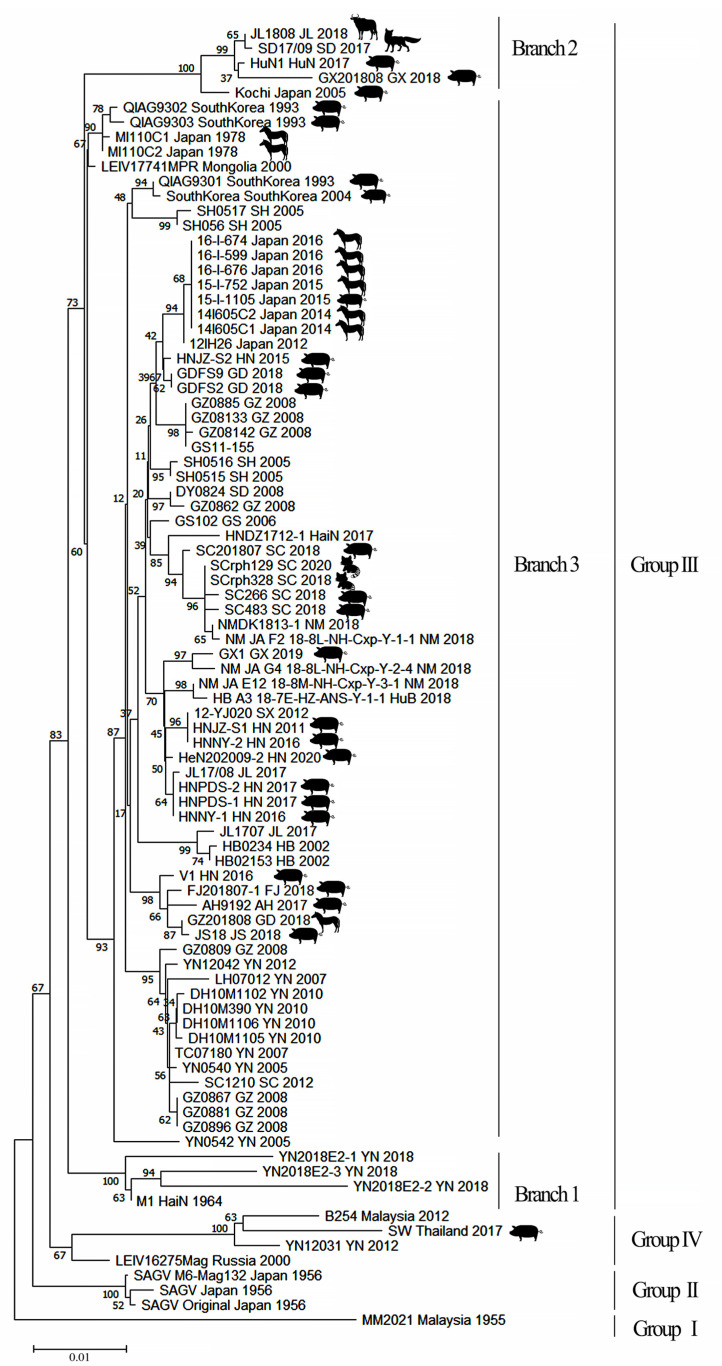
Phylogenetic analysis of GETV genes (E2). Using MEGA-X software, the neighbor-joining (NJ) method was used to draw the phylogenetic tree with 1000 bootstrap replicates. GETV can be divided into four groups (I–IV), of which group III can also be divided into three evolutionary branches (1–3). The animal host isolates are marked with different animal patterns on the figure and isolates without patterns were all isolated from mosquitoes.

Phylogenetic analysis of E2 gene sequence information for GETV in GenBank (virus isolated in 1955 to 2022 from bloodsucking insects and animal isolates) revealed four groups (GI–IV). Among them, only one strain of virus (MM2021) belonged to GI, which was isolated in Malaysia in 1955; GII included three strains of GETV (SAGV) isolated in Japan in 1956; GIV included four strains isolated from Thailand (SW), Yunnan, China (YN12031), Malaysia (B254) and Russia (LEIV/16275/Mag). Notably, the remaining 80 GETV isolates, whether isolated from bloodsucking insects or animal specimens, belonged to GIII (Table S1, Figure 1 and Figure 2).

The above results indicate that GI, GII and GIV of GETV have been present in low-latitude tropical regions over the past 70 years [48,49]. By contrast, the GIII GETV isolates are not only numerous (accounting for 90% (80/88) of all GETV isolates), but also has distinct characteristics. (1) GIII has a large isolation time span. The first GIII GETV strain (M1) was isolated in 1964, and the most recent GETV strain (HeN202009-2) was isolated in 2020. (2) GIII has spread from the tropic region to temperate regions. GIII GETV was isolated from 19° north latitude (Hainan Island in China) to 51° north latitude (Mongolia) [13,14]. (3) There are numerous species of mosquitoes carrying GIII GETV. GIII virus has been isolated from 17 species of mosquitoes in five genera, as well as midge vectors. It is suggested that GIII has a wider range of bloodsucking insect species that carry and spread the virus than other groups, consistent with the wide range of this group virus. (4) Phylogenetic analysis showed that GIII GETV can be further divided into three evolutionary branches. The branch 3 contains the highest number of viruses, as well as the most bloodsucking insects and animal isolates, ranging from low latitudes to high latitudes (Figure 2). (5) GIII GETV is the main group that causes animal diseases. Over the past 20 years, all viruses causing animal diseases are GIII isolates, such as Japanese horse isolates in 1978 [4], Japanese pig isolates in 2015 [38], Chinese pig isolates in 2017 [7] and Chinese blue fox isolates in 2017 [23] (Table 1). GIII has not only caused multiple disease outbreaks in horses and pigs, but, in recent years, GIII has also caused diseases in cattle [22], blue foxes [23] and red pandas [29].

In conclusion, compared to GI, GII and GIV, the GIII GETV has a wider variety of mosquitoes as transmission vectors and many more animals that act as virus amplification hosts. Additionally, the vast continent of Eurasia provides ideal conditions for the further expansion of the GIII virus. It is conceivable that GIII will not only become the dominant group of GETV but will also continue to expand its geographical scope. 

### 4.2. Evolutionary Dynamics and Migration of GETV

Analyses of the molecular evolution and virus migration route of GETV show that, in terms of the most recent common ancestor (tMRCA) of GETV, the virus originated ~145 years ago (95% HPD; 75–244) and gradually evolved into four groups. GI was the first GETV, isolated in Malaysia in 1955 (MM2021 strain). GII emerged ~60 years ago (95% HPD; 59–73), including SAGV isolated in Japan in 1956. GIII viruses appeared ~50 years ago (95% HPD; 51–72). GIII can be divided into two evolutionary branches; GETV (M1) isolated in China in 1964, the oldest virus in the GIII evolution branch, and other GIII viruses similar to M1 viruses that are completely independent and have evolved into a second GIII branch in parallel. GIV is the youngest group, emerging ~30 years ago (95% HPD; 16–55). Thus, GETV belongs to the class of emerging viruses [12,49].

Studies on the migratory transmission route of GETV show that Malaysia, Japan and Yunnan Province in China are the three main dissemination sites of this virus. GETV may have spread from Malaysia to Japan in 1917, from Japan to Hainan Province in China in the 1960s, from Japan to Yunnan Province in China in the 1970s, then to Hebei, Shanghai, Gansu and Sichuan provinces in China through Yunnan Province as the source of dissemination [49,50]. Combined with the results of molecular genetic evolution analysis of GETV, this implies that GI, GII and GIV have become a stable virus population prevalent in localized areas, whereas the geographical distribution of GIII has expanded rapidly, especially over the past 20 years, and has continued to spread northward in mainland Asia and Mongolia. The transmission and migration routes of GETV in continental Asia are similar to those of Japanese Encephalitis virus that is widely distributed in natural hosts in the region [51].

## 5. Vaccines

GETV infection can cause various animal diseases, and animal experiments suggest that the virus not only infects animals through mosquito bites, but can also be transmitted in horse herds through aerosols [52], causing abortion and stillbirth in pregnant pigs [53]. To reduce the risk of GETV infection in animals, Japanese scientists developed a formalin-inactivated GETV vaccine (MI-110 isolated from horses) [54,55].

Since 1979, thoroughbred racehorses have been vaccinated against GETV [54]. The vaccination program includes two doses of the vaccine for 2-year-old horses, at an interval of ~1 month, then a booster dose every year before summer (the peak mosquito period). Pleasingly, no further outbreaks of disease have been found in vaccinated herds. Subsequent findings have also shown that the vaccine may also be used for emergency vaccination of animals during epidemics [55]. However, in 2014, in the same training center (Miho Training Center) where the outbreak of GETV disease occurred in 1978, 75 of 2000 racehorses showed clinical signs of GETV infection and 33 febrile horses were positive for GETV infection by RT-PCR, NT or both. Most of the affected animals were 2-year-old horses that had received only one dose of the GETV vaccine. Further analysis found that sick horses included those that had been vaccinated throughout the whole process, suggesting that the currently used vaccine has a certain protective effect on horses, but cannot completely protect against infection by newly emerged strains [24].

Oil emulsion-inactivated vaccine prepared from a porcine GETV isolate (GETV-JS18) achieved 100% protection of tested pigs; the average neutralizing antibody tire after vaccine immunization reached 9.8 ± 0.8 Log2, and was maintained at a high level for at least 7 months [27].

## 6. Conclusions

Over the past 20 years, GETV has spread rapidly from low-latitude tropical regions to high-latitude temperate regions, causing disease outbreaks in animals in many countries in Asia. As a result, the gradual expansion of GETV and its infection from the Asian region of Eurasia to the European region became possible. Therefore, strengthening the detection and monitoring of GETV infection in Eurasia is essential to detect outbreaks in animals in a timely manner, and thereby reduce economic losses in animal husbandry. Additionally, although GETV has not yet been found to cause outbreaks of human disease, it is particularly important to monitor people who work in animal husbandry (especially those working with horses and pigs) in areas where GETV is endemic to avoid cross-species infection to humans. 

## Figures and Tables

**Table 1:** GETV disease outbreaks in animals.

Country	Region	Year	Animals				Group †	Reference
			Horse	Pig	Blue Fox	Cattle		
Japan	Kanto district, Sakai T.C.	1978	0/70/455 *					[20]
	Ibaraki Prefecture, Miho T.C.	1978	0/722/1903				III	[4]
	Tochigi Prefecture, Ritto T.C.	1979	0/136/-					[21]
	Kanagawa Prefecture, Pig farm	1985		8/12/–				[5]
	Ibaraki Prefecture, Miho T.C.	2014	0/75/2000				III	[24]
	Ibaraki Prefecture, Miho T.C.	2015	0/30/1992				III	[25]
India	Horse farm	1990	0/26/88					[6]
China	Hunan Province, pig farm	2017		200/1333/2915			III	[7]
	Shandong Province, animal farm	2017			6/25/–		III	[23]
	Guangxi Province, pig farm	2018		0/54/503			III	[10]
	Jilin Province, cattle farm	2018				0/10/48	III	[22]
	Guangdong Province, T.C.	2018	0/1/–				III	[26]

† The GETV group identified from the outbreaks. T.C., equestrian training center. * number of deaths/number of cases/total number of reared animals. –, no specific number of reared animals.

**Table 2:** Seroepidemiological analysis of GETV infection in animals.

Country	Region	Year	Method *	Animals				Reference
				Horse	Pig	Wild Boar	Cattle	
Japan	Hokkaido (Tokachi)	1973–1976	SNT	12.7 (8/127) **				[35]
	Hokkaido (Hidaka)	1976	SNT	11.4 (8/70)				
	Tokyo	1977	SNT	33.3 (17/51)				
	Hokkaido (Southern)	1979–1981	HIT	35.7 (107/300)				[36]
	Hokkaido (Central)	1979–1981	HIT	31.7 (57/180)				
	Hokkaido (Eastern)	1979–1981	HIT	7.5 (15/200)				
	Hokkaido (Northern)	1979–1981	HIT	45.7 (105/230)				
	Miho T.C. (Infected horses)	1978	SNT	93 (120/129)				[30]
	Miho T.C. (Uninfected horses)	1978	SNT	24.9 (52/152)				
	Miho T.C. (Before the GETV outbreak)	1978	SNT	6 (14/232)				
	Miho T.C. (After the GETV outbreak)	1978	SNT	61.2 (172/281)				
	Hokkaido	1978	SNT	52.2 (12/23)				
	Tohoku	1978	SNT	6.7 (1/15)				
	Kanto	1978	SNT	18.8 (36/192)				
	Chubu	1978	SNT	54.5 (55/101)				
	Kansai	1978	SNT	3.6 (5/140)				
	Kyushu	1978	SNT	72 (18/25)				
	Nakayama Racecourse	1972–1977	SNT	25.9 (2346/1338)				
	Hokkaido	1980	HIT		2.7 (1/37)			[31]
	Tohoku	1980	HIT		16.8 (20/119)			
	Kanto	1980	HIT		19.1 (40/209)			
	Chubu	1980	HIT		14.2 (36/254)			
	Chugoko	1980	HIT		11.5 (18/156)			
	Kinki	1980	HIT		7.1 (15/211)			
	Shikoku	1980	HIT		11 (13/118)			
	Kyushu	1980	HIT		14.8 (31/209)			
	Kyushu	2000–2001	HIT			47.8 (43/90)		[37]
	Shimonoseki	2013–2014	PRNT			54.3 (39/70)		[32]
	Nationwide	2007–2011	ELISA			3 (3/301)		
	Nationwide	2012	ELISA			15.5 (18/116)		
	Nationwide	2013	ELISA			44.6 (4/192)		
	Nationwide	2014	ELISA			29.1 (46/158)		
	Nationwide	2015	ELISA			18.1 (26/144)		
	Nationwide	2016	ELISA			14.3 (34/237)		
	Nationwide	2007–2016	ELISA			16.0 (168/1048)		
	South Ibaraki	2012	VNT		1 (1/100)			[38]
	South Ibaraki	2013	VNT		0 (0/97)			
	South Ibaraki	2014	VNT		28.8 (19/66)			
	South Ibaraki	2015	VNT		65 (77/117)			
	North Chiba	2010	VNT		1.6 (2/123)			
	North Chiba	2011	VNT		0 (0/111)			
	North Chiba	2012	VNT		0 (0/74)			
	North Chiba	2013	VNT		14.1 (19/135)			
	North Chiba	2014	VNT		17.8 (8/45)			
	North Chiba	2015	VNT		48 (24/50)			
South Korea	Nationwide	1985	HIT	37 (212/575)				[39]
	Nationwide	1985	HIT	47 (218/462)				
India	Nationwide	1990	SNT	17 (26/152)				[6]
Thailand	Eleven provinces	2017–2018	ELISA		23.1 (275/1188)			[40]
China	Yunnan †	2015	PRNT		45.88 (39/85)		71.88 (23/32)	[33]
	Xinjiang †	2017–2020	ELISA	70.2 (1140/1625)	51.1 (203/397)		25.1 (100/398)	[34]
	Hainan	1979	NT	25 (4/16)	17.6 (3/17)			[13]
	Hainan †	1982	CFT		22.7 (5/22)			
	Guangzhou	2019	SNT	26.09 (48/184)				[41]
	Yunnan	2019	ELISA		47.78 (43/90)			[42]
Indonesia	Java and Bali	1979	HIT				1.1 (1/90)	[43]
Malaysia	Sarawak	1962–1964	HIT		52 (205/395 )			[44]
	Sarawak	1962–1964	NT		77 (210/272)			

* Method: SNT, serum neutralization test; HIT, hemagglutination inhibition test; PRNT, plaque reduction neutralization test; ELISA, enzyme-linked immunosorbent assay; VNT, virus neutralization test; CFT, complement fixation test; NT, neutralization test. ** Positive rate of samples (%) (number of positive samples/total number of samples). † See text for description.

## Data Availability

Not applicable.

## Supplementary Materials

The following supporting information can be downloaded at: https://www.mdpi.com/article/10.3390/pathogens11080945/s1, Table S1: GETV isolates from bloodsucking insects and vertebrate hosts. As of March 2022, 169 GETV strains had been identified. Virus sequences 128 are from GenBank, including 88 E2 genes and 40 non-E2 genes (C, E1, nsP1–4). Information for 41 isolates is from published Chinese and English articles. A total of 113 strains were isolated from bloodsucking insect vectors, and the remaining 56 strains were isolated from animal specimens. References [14,15,16,17,18] are cited in the Supplementary Materials.
