# Supplementary material for: Getah Virus (Alphavirus): An Emerging, Spreading Zoonotic Virus
Source: Pathogens. 2022 Aug 20;11(8):945. doi: 10.3390/pathogens11080945 (PMC9416625; doi:10.3390/pathogens11080945)
Supplement: Supplementary file 1 [file pathogens-11-00945-s001.zip › pathogens-1840034-supplementary.pdf]

Table S1. GETV isolates from bloodsucking insects and vertebrate hosts. As of March 2022, 169 GETV strains had been identified. Virus sequences 128 are from GenBank, including 88 E2 genes and 40 non-E2 genes (C, E1, nspP1–4) . Information for 41 isolates is from published Chinese and English articles. A total of 113 strains were isolated from bloodsucking insect vectors, and the remaining 56 strains were isolated from animal specimens.

Group	Strain	Year	Country	source		Source of information		
						GenBank		Reference
				blood-sucking insects	animals	E2 gene	Not E2 gene	
I	MM2021	1955	Malaysia	*Cx. gelidus*		AF339484*		
II	GETV-SAGV	1956	Japan	mosquito		AB032553*		
II	GETV-SAGV-Original	1956	Japan	mosquito		AF339483		
II	GETV-SAGV-M 6-Mag 132	1956	Japan	mosquito		MW410934*		
IV	LEIV 16275 Mag	2000	Russia	*Ae. sp.*		EF631998*		
IV	YN12031	2012	China, Yunnan	*Ar. subalbatus*		KY434327*		
IV	B254	2012	Malaysia, Johor	*Cx. fuscocephalus*		LR990838*		
IV	SW_Thailand	2017	Thailand		Wild boar	LC534253*		
III	M1	1964	China, Hainan	*Cx. sp.*		EU015061*		
III	MI-110-C2	1978	Japan		Horse	LC079087*		
III	MI-110-C1	1978	Japan		Horse	LC079086*		
III	QIAG9303	1993	South Korea		Pig	KR081240		
III	QIAG9302	1993	South Korea		Pig	KR081239		
III	QIAG9301	1993	South Korea		Pig	KR081238		
III	LEIV 17741 MPR	2000	Mongolia	*Cx. sp.*		EF631999*		
III	HB0234	2002	China, Hebei	*Cx. tritaeniorhynchus*		EU015062*		
III	HB0215-3	2002	China, Hebei	*Cx. tritaeniorhynchus*		EU015065		
III	South Korea	2004	South Korea		Pig	AY702913*		
III	SH05-17	2005	China, Shanghai	*Cx. tritaeniorhynchus*		EU015069		
III	SH05-16	2005	China, Shanghai	*Cx. tritaeniorhynchus*		EU015068		

III	SH05-15	2005	China, Shanghai	*Cx. tritaeniorhynchus*		EU015067
III	SH05-6	2005	China, Shanghai	*Cx. tritaeniorhynchus*		EU015066
III	YN0540	2005	China,Yunnan	*Ar. subalbatus*		EU015063*
III	YN0542	2005	China, Yunnan	*Ar. subalbatus*		EU015064
III	Kochi/01/2005	2005	Japan		Wild boar	AB859822*
III	GS10-2	2006	China, Gansu	*Ar. subalbatus*		EU015070
III	TC07180	2007	China, Yunnan	*Cx. pseudovishnui*		KY450684
III	LH07012	2007	China, Yunnan	*Cx. tritaeniorhynchus*		KY450685
III	GZ0809	2008	China, Guizhou	*Ar. subalbatus*		KY457545
III	GZ0862	2008	China, Guizhou	*Ar. subalbatus*		KY457546
III	GZ0867	2008	China, Guizhou	*Ar. subalbatus*		KY457547
III	GZ0881	2008	China, Guizhou	*Ar. subalbatus*		KY457548
III	GZ0885	2008	China, Guizhou	*Ar. subalbatus*		KY457549
III	GZ0896	2008	China, Guizhou	*Ar. subalbatus*		KY457550
III	GZ08133	2008	China, Guizhou	*Ar. subalbatus*		KY457551
III	GZ08142	2008	China, Guizhou	*Cx. tritaeniorhynchus*		KY457552
III	DY0824	2008	China, Shandong	*Cx. tritaeniorhynchus*		KY434328
III	DH10M1102	2010	China, Yunnan	*Cx. fuscocephala*		KY450686
III	DH10M1106	2010	China, Yunnan	*Cx. annulus*		KY450687
III	DH10M390	2010	China, Yunnan	*Cx. tritaeniorhynchus*		KY450688
III	DH10M1105	2010	China, Yunnan	*An. Sinensis*		KY450689
III	HNJZ-S1	2011	China, Henan		Pig	KY363862*
III	12-YJ020	2012	China, Shanxi	mosquito		KP216576
III	SC1210	2012	China, Sichuan	*Ar. subalbatus*		LC107870*
III	YN12042	2012	China, Yunnan	*Cx. tritaeniorhynchus*		KY450683*
III	12IH26	2012	Japan	*Cx. tritaeniorhynchus*		LC152056*

III	14-I-605-C2	2014	Japan		Horse	LC079089*
III	14-I-605-C1	2014	Japan		Horse	LC079088*
III	HNJZ-S2	2015	China, Henan		Pig	KY363863*
III	15-I-1105	2015	Japan		Pig	LC212973*
III	15-I-752	2015	Japan		Horse	LC212972*
III	GETV-V1	2016	China, Henan		Pig	KY399029*
III	HNNY-2	2016	China, Henan		Pig	MG865967*
III	HNNY-1	2016	China, Henan		Pig	MG865966*
III	16-I-676	2016	Japan		Horse	LC223132*
III	16-I-674	2016	Japan		Horse	LC223131*
III	16-I-599	2016	Japan		Horse	LC223130*
III	HNDZ1712-1	2017	China,Hainan	mosquito		ON828424*
III	HNPDS-2	2017	China, Henan		Pig	MG865969*
III	HNPDS-1	2017	China, Henan		Pig	MG865968*
III	AH9192	2017	China, Anhui		Pig	MG865965*
III	HuN1	2017	China, Hunan		Pig	MF741771*
III	JL1707	2017	China, Jilin	mosquito		MH722255*
III	JL17/08	2017	China, Jilin	mosquito		MG869691*
III	SD17/09	2017	China, Shandong		Blue fox	MH106780*
III	SC266	2018	China, Sichuan		Pig	MN478487*
III	SC483	2018	China, Sichuan		Pig	MN478486*
III	NMDK1813-1	2018	China, Inner Mongolia	mosquito		MW512827*
III	JS18	2018	China, Jiangsu		Pig	MT210319*
III	FJ201807-1	2018	China, Fujian		Pig	MZ736799*
III	GDFS9	2018	China, Guangdong		Pig	MT086509*
III	GDFS2	2018	China, Guangdong		Pig	MT086508*

III	GZ201808	2018	China, Guangdong		Horse	MK487997*	
III	GX201808	2018	China, Guangxi		Pig	MT269657*	
III	HB_A3_18-7E-HZ-ANS-Y-1-1	2018	China, Hubei	*An. sinensis*		MW246754	
III	NM,JA_F2_18-8L-NH-Cxp-Y-1-1	2018	China, Inner Mongolia	*Cx. pipiens*		MW246769*	
III	NM,JA_G4_18-8L-NH-Cxp-Y-2-4	2018	China, Inner Mongolia	*Cx. pipiens*		MW246756	
III	NM,JA_E12_18-8M-NH-Cxp-Y-3-1	2018	China, Inner Mongolia	*Cx. pipiens*		MW246755	
III	GS11-155	2018	China, Gansu	*Cx. tritaeniorhynchus*		ON828425*	
III	JL1808	2018	China, Jilin		cattle	MH722256*	
III	SC201807	2018	China, Sichuan		Pig	MK693225*	
III	SCrph328	2018	China, Sichuan		*Ailurus fulgens*	MZ357111*	
III	YN2018E2-1	2018	China, Yunnan	mosquito		MZ494498	
III	YN2018E2-2	2018	China, Yunnan	mosquito		MZ494499	
III	YN2018E2-3	2018	China, Yunnan	mosquito		MZ494500	
III	GX1	2019	China, Guangxi		Pig	MZ736796*	
III	HeN202009-2	2020	China, Henan		Pig	MZ736801*	
III	SCrph129	2020	China, Sichuan		*Ailurus fulgens*	MZ357112*	
subtotal	88 strains			49 strains	39 strains		
	AHHB	2016	China, Anhui		Pig		MG817411
	JS,QH_G7_18-7L-QH-Cxt-5-4	2018	China, Hainan	*Cx. tritaeniorhynchus*			MW246722
	XY0901	2009	China, Henan	*Cx. tritaeniorhynchus*			JN582180
	XY0904	2009	China, Henan	*Cx. tritaeniorhynchus*			JN582181
	HNGY	2016	China ,Henan		Pig		MG817427
	HNKF	2016	China, Henan		Pig		MG817426
	HNXC	2016	China, Henan		Pig		MG817422

HNXY	2016	China, Henan		Pig	MG817419
HNBF-2	2016	China, Henan		Pig	MG817418
HNWG	2016	China, Henan		Pig	MG817413
HNZM	2016	China, Henan		Pig	MG817412
HNBF-1	2016	China, Henan		Pig	MG817409
HNHB	2016	China, Henan		Pig	MG817408
LN0636	2006	China, Liaoning	*An. sp.*		FJ217992
Z39	2008	China, Liaoning	*Cx. tritaeniorhynchus*		FJ897148
Z18	2008	China, Liaoning	*Cx. tritaeniorhynchus*		FJ897147
Z12	2008	China, Liaoning	*Cx. tritaeniorhynchus*		FJ897146
B1	2008	China, Liaoning	*Cx. tritaeniorhynchus*		FJ897145
SD2014-1	2015	China, Shandong		Fox	KX034052
YN08	2008	China, Yunan	*Ae. albopictus*		JN578105
SAVG_ML/Taiwan/02	2002	China, Taiwan		Pig	DQ138299
SAVG_Taiwan	2002	China, Taiwan	mosquito		AF242890
A87-09	2009	China, Yunan	mosquito		KP900945
A137-09	2009	China, Yunan	mosquito		KP900944
A134-09	2009	China, Yunan	mosquito		KP900943
A132-09	2009	China, Yunan	mosquito		KP900942
A123-09	2009	China, Yunan	mosquito		KP900941
YN2018NS3-2	2018	China, Yunan	mosquito		MZ494502
YN2018NS3-1	2018	China, Yunan	mosquito		MZ494501
GETV-B3	2018	China, Yunan	*Cx. tritaeniorhynchus*		MW030467
GETV-C1	2018	China, Yunan	*Ae. aegypti*		MW030466
GETV-E1	2018	China, Yunan	*Cx. tritaeniorhynchus*		MW030465
XJ-2017-08	2017	China, Xinjiang		Pig	OK626897

	XJ-2018-06	2018	China, Xinjiang		Horse	OK626898	
	XJ-2019-06	2019	China, Xinjiang		Pig	OK626899	
	SAVG_31407	1956	Japan	mosquito		AF398379	
	Miho2014	2014	Japan		Horse	LC012885	
	KorS1010	2010	South Korea	*Ae. vexans nipponii*		JN410945	
	KorL915	2010	South Korea	*Ae. vexans nipponii*		JN410944	
	Jin-Ju	2010	South Korea	mosquito		JN410946	
subtotal	40 strains			24 strains	16 strains		
	N544	1961	Australia	mosquito			[1]
	–	1966	Cambodia	*Cx. tritaeniorhynchus*			[1]
	XJ73	2016	China, Xinjiang	*culicoides*			[14]
	SZC30	2013	China, Yunnan	*culicoides*			[15]
	Ph Ar 814	1977	Philippines	*Cx. vishnui*			[16]
	An-380	1986	Sri Lanka	*C. gelidus*			[17]
	An-457	1986	Sri Lanka	*Cx. tritaeniorhynchus*			[17]
	An-514	1986	Sri Lanka	*Cx. tritaeniorhynchus*			[17]
	K-348	1986	Sri Lanka	*Cx. tritaeniorhynchus*			[17]
	An-432	1986	Sri Lanka	*Cx. fuscocephala*			[17]
	A-833	1987	Sri Lanka	*Cx. tritaeniorhynchus*			[17]
	A-836	1987	Sri Lanka	*Cx. tritaeniorhynchus*			[17]
	A-890	1987	Sri Lanka	*Cx. tritaeniorhynchus*			[17]
	A-989	1987	Sri Lanka	*Cx. tritaeniorhynchus*			[17]
	–	1990	India		Horse		[6]
	GETV 1	2004	Vietnam	*An. agus*			[18]
	GETV 2	2004	Vietnam	*An. vagus*			[18]
	GETV 3	2004	Vietnam	*An. vagus*			[18]

	GETV 4	2004	Vietnam	*Cx. bitaeniorhynchus*		[18]
	GETV 5	2004	Vietnam	*Cx. tritaeniorhynchus*		[18]
	GETV 6	2004	Vietnam	*Cx. tritaeniorhynchus*		[18]
	GETV 7	2004	Vietnam	*Cx. tritaeniorhynchus*		[18]
	GETV 8	2004	Vietnam	*Cx. tritaeniorhynchus*		[18]
	GETV 9	2004	Vietnam	*Cx. vishnui*		[18]
	GETV 10	2004	Vietnam	*Cx. vishnui*		[18]
	GETV 11	2004	Vietnam	*Cx. vishnui*		[18]
	GETV 12	2004	Vietnam	*Cx. vishnui*		[18]
	GETV 13	2004	Vietnam	*Cx. vishnui*		[18]
	GETV 14	2004	Vietnam	*Cx. vishnui*		[18]
	GETV 15	2004	Vietnam	*Ma. indiana*		[18]
	SAVG 1	2004	Vietnam	*An. sinensis*		[18]
	SAVG 2	2004	Vietnam	*An. vagus*		[18]
	SAVG 3	2004	Vietnam	*An. vagus*		[18]
	SAVG 4	2004	Vietnam	*An. spp.*		[18]
	SAVG 5	2004	Vietnam	*Ar. subalbatus*		[18]
	SAVG 6	2004	Vietnam	*Cx. gelidus*		[18]
	SAVG 7	2004	Vietnam	*Cx. gelidus*		[18]
	SAVG 8	2004	Vietnam	*Cx. tritaeniorhynchus*		[18]
	SAVG 9	2004	Vietnam	*Cx. tritaeniorhynchus*		[18]
	SAVG 10	2004	Vietnam	*Cx. vishnui subgroup*		[18]
	SAVG 11	2004	Vietnam	*Ma. annulifera*		[18]
subtotal	41 strains			40 strains	1 strains	
total	169 strains			113 strains	56 strains	

* Complete genome sequence
